# Is Estrogen a Curse or a Blessing in Disguise? Role of Estrogen in Gastroesophageal Reflux Disease

**DOI:** 10.7759/cureus.11180

**Published:** 2020-10-26

**Authors:** Ayesha Kang, Rhutuja Khokale, Oluwatayo J Awolumate, Hafsa Fayyaz, Ivan Cancarevic

**Affiliations:** 1 Internal Medicine, California Institute of Behavioral Neurosciences & Psychology, Fairfield, USA; 2 Neurology, California Institute of Behavioral Neurosciences & Psychology, Fairfield, USA; 3 Family Medicine, California Institute of Behavioral Neurosciences & Psychology, Fairfield, USA

**Keywords:** gastroesophageal reflux disease, estrogen, pathophysiology, hormonal replacement therapy, gender differences, pregnancy, heartburn, quality of life

## Abstract

Gastroesophageal reflux disease (GERD), a condition wherein there is reflux of stomach contents into the esophagus, causing heartburn and regurgitation with a sour and bitter taste in the mouth. It may or may not lead to mucosal injury. GERD symptoms can be troublesome and negatively impact the quality of life. Estrogen, the sex hormone in females, may play a role in the gender differences observed in GERD symptoms. This review article analyzes estrogen's mechanism in the causation of GERD symptoms and its complications. A better understanding of pathophysiology will help us guide early detection, treatment, and prevention of repeated reflux complications. We did a comprehensive PubMed database search and analyzed differences in GERD symptoms experienced by males and females and the role of estrogen in erosive and non-erosive GERD. GERD symptoms in association with hormonal replacement therapy (HRT) and pregnancy, the lower esophageal sphincter (LES) relaxant effects, and estrogens' protective effect on the esophagus from mucosal injury due to repeated reflux are discussed. Estrogen can cause GERD as an adverse effect and, at the same time, can be used to protect the mucosa from GERD induced injury and its complications like metaplasia and cancer. The mechanism is complex and requires further studies and trials. We recommend future researchers to look for possible estrogen use to treat erosive GERD and complication prevention.

## Introduction and background

Gastroesophageal reflux disease (GERD) is the backflow of stomach content into the esophagus leading to a sour and bitter taste in the mouth [1,2]. Millions of people are affected worldwide, with a prevalence of 18.1%-27.8% in North America [2]. Montreal criteria from 2006 defined GERD as a backflow of stomach material that causes bothersome symptoms of heartburn and regurgitation and subsequent complications that may or may not lead to esophageal mucosal injury [1]. GERD etiology is multifactorial; one of the most common causes is disruption at the gastroesophageal junction, either via relaxation of the lower esophageal sphincter (LES) or increased intra-abdominal pressure, which promotes the backflow into the esophagus [2,3]. It is a relatively benign condition but significantly impacts the quality of life [4]. 

Estrogen is a female sex steroidal regulatory hormone that plays a role in GERD symptoms. Estrogen receptors present in the gastrointestinal epithelium are involved in GI diseases' pathophysiology, including GERD [5]. Estrogen works via its alpha and beta receptors and increases nitric oxide synthesis, a notorious muscle relaxant, decreasing smooth muscles' tone in the lower esophageal sphincter [5-8]. In females that used estrogen replacement therapy (ERT), the risk of GERD symptoms is increased by 32% [8]. 

During pregnancy, GERD symptoms increase to a prevalence of 30-80% [9,10]. A prospective longitudinal cohort study that compared pregnant and nonpregnant females reports significantly increased GERD symptoms [10]. Several mechanisms contribute to GERD symptoms in pregnancy, including decreased LES pressure, increased intra-abdominal pressure due to the enlarging uterus, and GI motility changes [11]. Increased circulating levels of progesterone and estrogen during pregnancy increases LES relaxation and allows reflux [12-14]. 

We aim to evaluate the association between estrogen and GERD. We reviewed the mechanism behind estrogen and GERD symptoms and complications. A better understanding of the pathophysiology regarding gender differences will help early therapy and prevent subsequent complications and morbidity and measures for improving life quality. Several ongoing studies on different mechanisms lead to GERD symptoms and how estrogen plays a role. We will be reviewing already published literature to find the association between estrogen and GERD and its complications and study its pathophysiology. We will also be comparing gender differences in GERD and estrogen's association with pregnant and postmenopausal females on hormone replacement therapy (HRT) in causing GERD. Will a better understanding of the association help us get closer to manage GERD better and improve the quality of life?

For this review article, the PubMed database was mainly used for the literature search to extract useful articles from the last 10 years. Keywords used were: GERD, estrogen, pathophysiology, HRT, gender differences, pregnancy, heartburn, quality of life. At first, we found articles generally related to GERD, then we searched for articles that studied estrogen and GERD association and the gender differences in GERD and its complications. We made sure to properly cite statistical data and new information used in this review article.

## Review

Gastroesophageal reflux disease and gender

GERD-specific symptoms affect males and females differently. It is essential to understand the gender differences in GERD symptoms as it can help us better understand the mechanism, prevention, and therapy. GERD can lead to erosive esophagitis and non-erosive reflux esophagitis (NERD). Kim et al. studied sex and gender differences in GERD and found that NERD is more common in females who present more with GERD symptoms than males. Furthermore, males develop erosive esophagitis and complications such as Barrett’s esophagus and cancer more often than females [15]. Estrogen could be related to gender differences, but more research needs to be done to address the potential confounders such as gender differences in alcohol and tobacco consumption. Jung et al. found several studies in their review of population-based studies, which shows no difference in the prevalence of GERD symptoms in males and females [16]. They found a study that performed a survey in 2003 of age 20-95 years old in Olmsted County that showed no sex difference in the prevalence of GERD between men (15%; 95% confidence interval [CI], 12.9-17.3%) and women (14%; 95% CI, 12-16%) [16]. However, the endoscopy based studies found that esophagitis was more common in males than in females [17]. A meta-analysis conducted by Cook et al. concluded that more males suffer pathological changes after reflux than females. They highlighted the increasing male/female ratio in progression to reflux complications-Barrets’ esophagus and esophageal carcinoma. The Barretts’ esophagus meta-analysis gave an overall pooled male/female sex ratio of 1.96 (95% CI, 1.77-2.17). For erosive reflux disease (ERD), the pooled male/female sex ratio was 1.57 (95% C,: 1.40-1.76) and, for non-ERD, 0.72 (95% CI, 0.62-0.84) [18]. Similarly, Ford et al. reviewed studies that found NERD is more common in females than in males with progressively increasing symptoms and frequency [19]. 

Lin et al. studied 543 adults with signs and symptoms of GERD. Pre-testing questionnaire assessment was done, and then patients were categorized and graded according to their extent and severity via endoscopic findings, ambulatory pH, and motility findings. Comparison among 341 men and 202 women was made, results showed that heartburn without esophagitis was noted in 38% of men and 55% of women patients. They also noted that women are less likely to develop Barrett’s esophagus (p<0.005) but revealed higher symptom severity for heartburn (p<0.01) as compared to men. This will help with early disease recognition and management [20].

**Figure 1:**
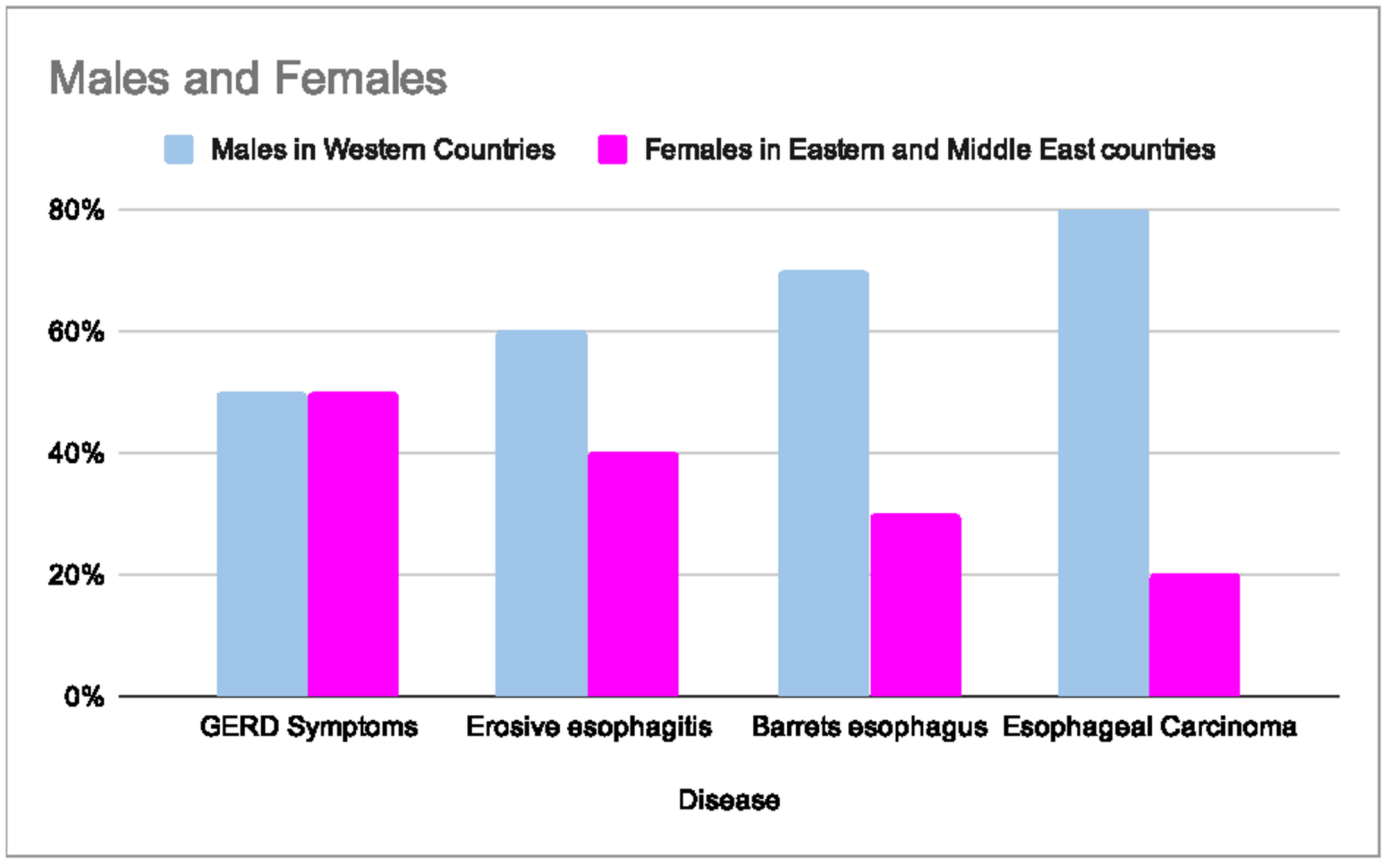
Distribution of the Gastroesophageal Reflux Disease and related diseases in males from Western Countries and females from Eastern and Middle East countries Adapted from Boeckxstaens et al. [21] Gastroesophageal Reflux Disease (GERD)

The distribution of GERD related diseases concerning gender differences varies greatly (Figure 1). Boeckxstaens et al. studied gender differences in GERD related disease. The data shows GERD complications being more common in men, but GERD symptoms incidence were equal in both males and females [21].

GI mucosal epithelium is a protective barrier that maintains the integrity and prevents injury [22]. In males and postmenopausal females, the mucosa is more susceptible to damage because of decreased estrogen (E2) levels [23]. Estrogen works via its alpha and beta receptors, present in epithelial cells, muscle cells, and immune cells [5,24]. The mechanism is that the E2 maintains the tight junctions (TJ) present in the epithelial cells [25]. Estrogen upregulates the occludin protein, which is a component of TJ [26]. So, the decreased levels of E2 in postmenopausal females and males affect these junctions and make the membrane more permeable to damaging agents and lead to mucosal injury [27]. 

For this reason, the epithelium will become more susceptible to GERD induced injury and subsequent complications, for example, esophagitis seen in these population groups. We can also deduce that females will have a later onset of GERD causing mucosal damage and cancer incidence. The anti-inflammatory function of estrogen plays a role in this incidence [28]. 

There were only limited articles available that studied gender differences in GERD and how estrogen plays a role. Asanuma et al. reviewed gender differences in GERD and estrogen’s role, a beneficial study for our review article. Similarly, in their study, they found a positive association between estrogen and gender differences in GERD symptoms. In postmenopausal females, the erosive GERD spectrum rapidly increased compared to reproductive age females; men had high rates of erosive GERD and its complications [28]. Estrogen is protective via its anti-inflammatory effects, but it still leads to an increased incidence of non-erosive GERD symptoms in younger females via its LES relaxation mechanism [5-7,28]. Table 1 demonstrates the incidence of erosive and non-erosive esophagitis among males and females.

**Table 1:** Gender differences in erosive and non erosive esophagitis Adapted from Richter et al. [30]

	Country	Males	Females	References
Erosive Esophagitis	US	4092	2617	[29]
	US	131	63	[21]
Non erosive Esophagitis	US	375	523	[30]
	US	73	150	[31]

Masaka et al. demonstrated estrogen’s anti-inflammatory action in a study on rats that shows estrogen reduces injury caused by erosive reflux through inactivation of the mast cells [32]. Kim et al. also studied gender-specific differences in GERD. They conducted a health retrospective cohort of 10,158 Korean men and women who had the screening done via self-reported questionnaires and upper endoscopy. They found that reflux esophagitis was more common in men than in women (10.6% vs. 2.0%, p< 0.001); and symptomatic GERD was more common in women (6.2% vs. 2.5%, p<0.001) [33]. 

Both studies by Kim et al. and Asanuma et al. found similar estrogen relationship findings in causing a spectrum of GERD symptoms and esophagitis in males versus females [15,28]. We can notice that both studies reported the protective effect of estrogen in mucosal injury. Clinically females had more incidence of NERD symptoms but erosive GERD and its progression towards esophagitis and subsequent metaplasia and cancer are more common in males [15,28]. Kim et al. study results supported our discussion of esophagitis being more common in males and symptomatic GERD in females [33]. We have reviewed how estrogen plays a role in gender differences in the GERD spectrum, but its mechanism is an active research area.

Gastroesophageal reflux disease and hormonal replacement therapy

Estrogen and progesterone both are components of HRT. Estrogen increases nitric oxide, which relaxes LES. A study conducted by Piccinini et al. also supports the similar mechanism of nitric oxide synthesis in females on estrogen replacement therapy (ERT) [34]. Hence, there is a positive correlation between GERD and hormonal replacement therapy (HRT) [6]. In females who used ERT, the risk of GERD symptoms is increased by 32% [9]. A retrospective cohort study conducted by Close et al. also found a statistically significant association of GERD and HRT with an odds ratio (OR) of 1.49; 1.18-1.89 [35]. They picked 51,182 menopausal women using the UK General Practice Research Database between 1995 to 2004; out of those, 8,831 were matched with and without hormone use. They adjusted the participants according to age, medicine use, and co-morbidities and found a statistically significant association between estrogen-only treatment and GERD symptoms (OR 1.49; 1.18-1.89) [35]. 

Jacobson et al. studied postmenopausal hormone use and symptoms of GERD. In their prospective cohort study of 51,637 postmenopausal women enrolled in the Nurses’ Health Study who provided data on the use of hormone therapy since 1976 and self-reported gastroesophageal reflux symptoms in 2002 [36]. The result showed 12,018 women reported reflux symptoms, which is 23% with an odds ratio of 1.46 (95% CI 1.36-1.56) for prior HRT users, 1.66 (95% CI 1.54-1.79) for current estrogen users, and 1.41 (95% CI 1.29-1.54) for current HRT users. The symptoms increased with increasing estrogen dose and duration (p<0.001) [36]. The mechanism is that the estrogen increases nitric oxide (NO) and relaxes smooth muscles in LES [5-7]. Nilsson et al. found a positive correlation [6]. Best et al. studied the effect of estrogen hormonal therapy on plasma NO and endothelin 1 levels in postmenopausal women. They found that estrogen use increases the plasma NO levels [37]. 

Menon et al. studied the relationship between women on hormonal replacement therapy and esophageal adenocarcinoma development. They found that prolonged duration of HRT is associated inversely with esophageal adenocarcinoma development [38]. The reason is estrogen’s anti-inflammatory action, which protects the GI epithelium from erosive GERD injury from repeated reflux. HRT use in postmenopausal women can lead to this protective effect by decreasing esophagitis and subsequent complications like Barrett’s metaplasia and cancer [38]. They found a statistically significant case-control cohort study done in the UK that showed decreased cancer risk with HRT after five to 10 years of use (Hazard Ratio [HR] 0.25; 95% CI, 0.07-0.95) [38]. A meta-analysis, done by Green et al. found a similar association (relative risk [RR], 0.68; 95% CI, 0.55-0.84; p<0.001) [39]. 

The evidence from our findings shows that the estrogen and incidence of GERD symptoms are positively associated. It can be considered as an adverse effect in women on postmenopausal hormone therapy. Due to estrogens’ protective effect on GI malignancies, could it be considered as a therapy? The mechanism is still so vast and complex that it requires active research.

Gastroesophageal reflux disease and pregnancy 

As many as 83.4% of females experience GERD symptoms during pregnancy [40]. GERD in pregnancy is multifactorial due to decreased LES pressure caused by female sex hormones, increased intraabdominal pressure, and decrease GI motility [12,13]. Malfertheiner et al. performed a prospective longitudinal cohort study on 510 pregnant females using reflux disease questions (RDQ) [10]. They found that the prevalence of GERD incidence increases from the first trimester to the 3rd trimester from 26.1% to 51.2% [10]. Van Thiel et al. studied progesterone and estrogen levels increase in the plasma as the pregnancy progresses, and LES pressures are decreased throughout [13]. The primary mechanism is LES pressure relaxation, and other factors like increased intra-abdominal pressure play a minor role in GERD symptoms during pregnancy [14]. 

Overall, from the studies’ findings, we can say that the frequency of GERD symptoms increases in pregnancy. We have also seen positive associations from the above studies that estrogen plays a vital role in its pathophysiology. Although GERD is a benign condition, it can significantly impact the quality of life during pregnancy. Therefore, it needs to be managed to provide maximum comfort to patients.

Gastroesophageal reflux disease and impact on the quality of life

GERD symptoms can be bothersome and impact the quality of life. The management of GERD can be challenging, considering the multifactorial nature of the disease. Tack et al. performed a systematic review in which they compared nineteen studies. They found that disruptive GERD significantly impacts life quality, leading to poor sleep, impaired physical and mental health [41]. Another systematic review by Becher et al. studied the similar association of GERD symptoms on health-related quality of life [42]. They had similar findings that GERD symptoms negatively impact physical and mental health [42]. Maleki et al. studied the quality of life with GERD in Iran [43]. They enrolled cases and controls to compare the impact, using questionnaires whose results showed the more inferior quality of life in patients with GERD than controls with a p-value of less than 0.001 [43]. 

Overall, from the reviews of studies, we can conclude that patients with GERD symptoms have a lower quality of life. Therefore, proper detection and management are essential in alleviating the symptoms to improve the quality of life. Management of GERD can be quite challenging as multiple factors are responsible for the disease. 

This review article attempts to clear the association between estrogen with GERD symptoms and its complications. The article highlights the importance of prevention and speedy treatment of the complications by establishing GERD and estrogen association. However, this article’s review is limited by available data; only full-text articles available over the past 10 years were used. If estrogen has its protective effect in preventing cancer, future researchers can provide more evidence that estrogen use is safe in preventing cancer.

## Conclusions

Estrogen plays a vital role in the pathophysiology of GERD symptoms. Females tend to experience GERD symptoms more than males, but males have more GERD pathological complications than females. These gender differences could be related to estrogen. However, there is a strong possibility of confounders like alcohol and tobacco. More research needs to be done to address potential confounders such as gender differences in alcohol and tobacco consumption and co-morbidities like obesity. Through the literature review, we were better able to understand the estrogen’s pathophysiology in erosive and non-erosive GERD. GERD symptoms can be bothersome and impact the quality of life. The management of these symptoms can be challenging, considering the multifactorial etiology. The role of estrogen in GERD symptoms is a complex field, and its mechanism is still an active area of research. Acknowledging estrogen’s protective effect on GI epithelial injury, will we be able to treat GERD with estrogen to prevent complications? 
